# BRCA1-IRIS Overexpression Promotes Formation of Aggressive Breast Cancers

**DOI:** 10.1371/journal.pone.0034102

**Published:** 2012-04-12

**Authors:** Yoshiko Shimizu, Hugh Luk, David Horio, Penelope Miron, Michael Griswold, Dirk Iglehart, Brenda Hernandez, Jeffrey Killeen, Wael M. ElShamy

**Affiliations:** Cancer Institute and Department of Biochemistry, University of Mississippi Medical Center, Jackson, Mississippi, United States of America; University of South Alabama, United States of America

## Abstract

**Introduction:**

Women with HER2^+^ or triple negative/basal-like (TN/BL) breast cancers succumb to their cancer rapidly due, in part to acquired Herceptin resistance and lack of TN/BL-targeted therapies. BRCA1-IRIS is a recently discovered, 1399 residue, *BRCA1* locus alternative product, which while sharing 1365 residues with the full-length product of this tumor suppressor gene, BRCA1/p220, it has oncoprotein-like properties. Here, we examine whether BRCA1-IRIS is a valuable treatment target for HER2^+^ and/or TN/BL tumors.

**Methodology/Principal Findings:**

Immunohistochemical staining of large cohort of human breast tumor samples using new monoclonal anti-BRCA1-IRIS antibody, followed by correlation of BRCA1-IRIS expression with that of AKT1, AKT2, p-AKT, survivin and BRCA1/p220, tumor status and age at diagnosis. Generation of subcutaneous tumors in SCID mice using human mammary epithelial (HME) cells overexpressing TERT/LT/BRCA1-IRIS, followed by comparing AKT, survivin, and BRCA1/p220 expression, tumor status and aggressiveness in these tumors to that in tumors developed using TERT/LT/Ras^V12^-overexpressing HME cells. Induction of primary and invasive rat mammary tumors using the carcinogen *N*-methyl-*N*-nitrosourea (NMU), followed by analysis of rat *BRCA1-IRIS* and *ERα* mRNA levels in these tumors.

High BRCA1-IRIS expression was detected in the majority of human breast tumors analyzed, which was positively correlated with that of AKT1-, AKT2-, p-AKT-, survivin, but negatively with BRCA1/p220 expression. BRCA1-IRIS-positivity induced high-grade, early onset and metastatic HER2^+^ or TN/BL tumors. TERT/LT/BRCA1-IRIS overexpressing HME cells formed invasive subcutaneous tumors that express high AKT1, AKT2, p-AKT and vimentin, but no CK19, p63 or BRCA1/p220. NMU-induced primary and invasive rat breast cancers expressed high levels of rat *BRCA1-IRIS* mRNA but low levels of rat *ERα* mRNA.

**Conclusion/Significance:**

BRCA1-IRIS overexpression triggers aggressive breast tumor formation, especially in patients with HER2^+^ or TN/BL subtypes. We propose that BRCA1-IRIS inhibition may be pursued as a novel therapeutic option to treat these aggressive breast tumor subtypes.

## Introduction

Apoptosis evasion increases cancer cells' chances to encounter further transforming mutations that can lead to resistance to therapy and/or disease progression [1], [2]. Apoptosis resistant cells often loose expression of tumor suppressors, such as p53 [3], [4], which is mutated in ∼50% of breast cancers, or gain expression of oncogenes such as AKT, which is overexpressed in ∼40% of breast cancers [5]. Part of AKT ability to induce malignant tumor progression and chemo-drug resistance lies in its ability to enhance expression of pro-survival proteins, e.g., survivin [5]–[8].

HER2 is a tyrosine kinase surface receptor belonging to the epidermal growth factor receptor family, which includes HER1 (*aka*, EGFR), HER3 and HER4 [9], [10]. The HER2 gene is located on chromosome 17 and encodes 185 kDa protein [9], [11]. EGFR or HER2 knockout mice show attenuated lobular structures and milk production, suggesting a role in postnatal development of the breast [9], [12]. HER family members dimerize to form functional receptors that are stimulated by auto-phosphorylation and then phosphorylate/activate a wide range of intracellular signaling cascades [9], [13]. Alterations in the HER family members have been detected in many cancers, including breast cancer [9], [14]. HER2 amplification is observed in 15–30% of breast cancer cases and is often associated with poor prognosis [9], [14]. Herceptin (*aka*, trastuzumab) is an effective neutralizing monoclonal antibody to HER2 that blocks it's signaling and thus function [9], [15], [16]. Therefore, HER2 amplification is also a predictive factor for response to systemic therapy [9], [17], [18].

The triple negative/basal-like (TN/BL) breast cancer subtype comprises ∼15% of all breast cancers and is defined as estrogen (ERα)-, progesterone (PR)- and HER2- (not amplified) receptor negativity, but basal-associated markers- (e.g., cytokeratin 5/6 and 17) positivity [19]. TN/BL tumors is associated with a poor prognosis and while they are responsive to a wide range of chemotherapeutic agents, the majority of patients relapse quickly with a visceral metastases including lung, liver and brain metastasis. Based on gene expression arrays data, the molecular features of TN/BL breast cancers often overlap with those of BRCA1-assocaited tumors.

We recently discovered BRCA1-IRIS, a 1399 residue BRCA1 locus product [20]. Its mRNA contains an uninterrupted open reading frame that extends from codon 1 of the BRCA1/p220 reading frame in exon 2 to the end of exon 11. It then continues in-frame for 34 more triplets into intron 11 where it terminates, hence the name ***I***n-frame ***R***eading of BRCA1 ***I***ntron 11 ***S***plice variant (IRIS). Although it and the full-length product of this locus, the tumor suppressor BRCA1/p220 [21] share 1365 residues, unlike BRCA1/p220, BRCA1-IRIS possesses oncogenic functions. For example, BRCA1-IRIS overexpression induces over-replication by inhibiting geminin negative function at DNA replication origins [20], and over-proliferation by up-regulating cyclin D1 expression [22], [23]. BRCA1-IRIS overexpression also induces resistance to apoptosis induced by chemo-, geno-, and cell-toxic stresses in human mammary (HME) and ovarian (HOSE) epithelial cells by inhibiting p53 and/or enhancing AKT and survivin expression and activation [24], [25]. Thus generating cells resistant to chemotherapeutic agents, such as etoposide, ionizing or UV-radiation, and oxidative stresses [24], [25].

Together, these data suggest that part of BRCA1-IRIS oncogenic function lies in its ability to promote formation of apoptosis resistance and thus aggressive tumor cells. To confirm that *in vivo* and to investigate the oncogenic role of BRCA1-IRIS in details, we used three different approaches. First, we immunohistochemically stained and analyzed a large cohort of primary breast tumor samples using a newly generated BRCA1-IRIS monoclonal antibody. We found that BRCA1-IRIS is overexpressed in the majority of breast tumors analyzed, especially those of the HER2^+^ and TN/BL subtypes. BRCA1-IRIS-positive tumors were high-grade, aggressive and metastatic tumors that expressed higher levels of AKT and survivin, and lacked expression of BRCA1/p220 compared to BRCA1-IRIS-negative tumors. Second, we analyzed subcutaneous xenografts tumors developed by HME cells overexpressing TERT/SV40 large T-antigen (LT)/BRCA1-IRIS or /Ras^V12^ in SCID mice. We found that TERT/LT/BRCA1-IRIS-induced (hereafter BRCA1-IRIS-induced) tumors were more invasive and showed increase expression of AKT and survivin when compared to TERT/LT/Ras^V12^-induced (hereafter Ras^V12^-induced, see [26]) tumors. Third, we analyzed primary as well as invasive breast tumors generated in rats following exposure to *N*-methyl-*N*-nitrosourea (NMU). We found that carcinogen-induced rat breast cancers overexpress rat *BRCA1-IRIS* mRNA in some aggressive primary tumors or upon disease progression. Collectively, BRCA1-IRIS overexpression appears to promote formation of aggressive, invasive and/or metastatic breast cancers and implies that inhibiting BRCA1-IRIS expression and/or activity could be pursued as a novel therapeutic option to treat breast cancer patients, especially those with HER2^+^ and/or TN/BL diseases.

## Results

### Generation of immunohistochemical grade mouse monoclonal anti-BRCA1-IRIS antibody

**Figure 1:**
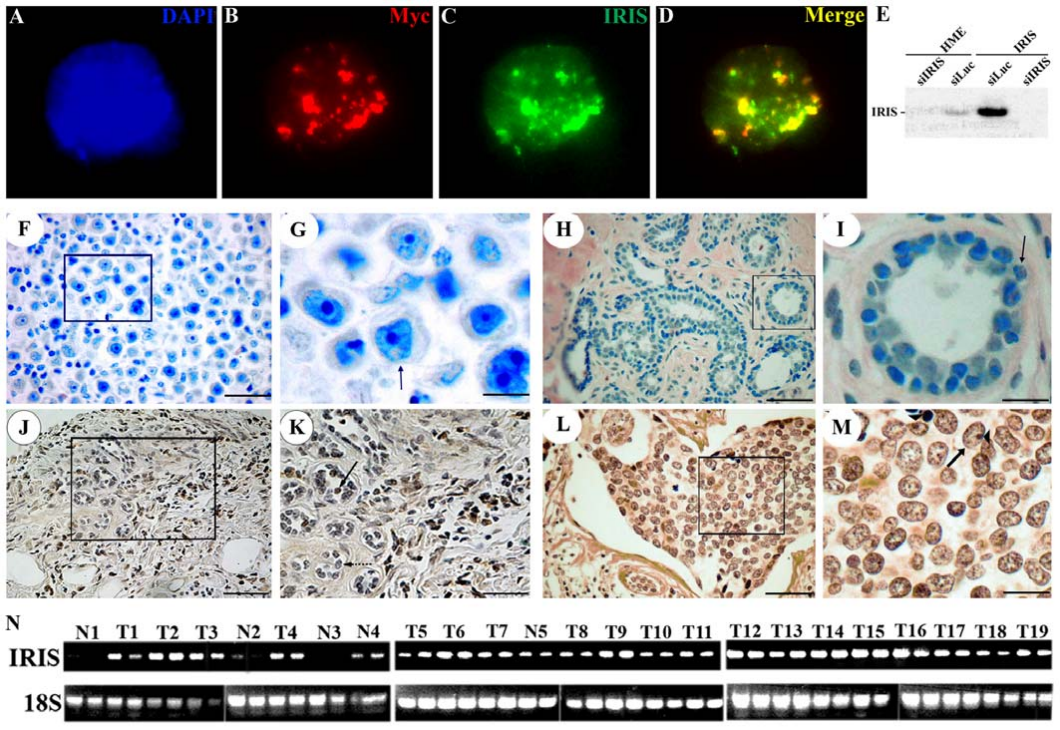
Expression of BRCA1-IRIS in breast tumor samples. (A) DAPI stained HME cells transfected with Myc-tagged BRCA1-IRIS cDNA. Same cell stained with anti-Myc (red, B), anti-BRCA1-IRIS (green, C). (D) Merge of B and C. (E) Expression of BRCA1-IRIS in HME or BRCA1-IRIS overexpressing cells (IRIS) following transfection of luciferase or BRCA1-IRIS siRNA. (F and G) are low and high magnification images of invasive breast cancer section stained with pre-absorbed anti-BRCA1-IRIS antibody. Expression of BRCA1-IRIS in paraffin embedded normal mammary epithelial tissue (H and I), DCIS (J and K) or invasive breast cancer tissues (L and M). H, J and L are low magnification images, whereas I, K and M are high magnification images. Note the BRCA1-IRIS-psoitive (solid arrow) and -negative (dashed arrow) cells in K. Also note that while all sections are counter stained with hematoxylin, for technical reasons, the staining was done for shorter time in J, K, L and M compared to F, G, H and I. (N) Expression of 18S and BRCA1-IRIS mRNA (in duplicates) in 5 normal (N) and 19 breast tumors (T). Bars are, 400 µm in H, J, 200 µm in F, K and L, 100 µm in I and M and 50 µm in G.

To study BRCA1-IRIS expression *in vivo*, a mouse anti-BRCA1-IRIS monoclonal antibody that recognizes an epitope in BRCA1-IRIS intron 11 was developed. The specificity of this antibody was validated by three separate approaches. 1) Double immunostaining of HME cells transfected with Myc-tagged BRCA1-IRIS cDNA (for 48 h) with anti-Myc tag antibody (9E10) and this anti-BRCA1-IRIS antibody. In these cells identical staining pattern was detected (Figure 1A–D). 2) Transfection of parental or BRCA1-IRIS overexpressing HME cells (hereafter IRIS) with luciferase or BRCA1-IRIS specific siRNA (against intron 11 [20]) for 72 h. In these cells, this antibody detected clear difference in BRCA1-IRIS expression in isogenic cells. Indeed, low endogenous BRCA1-IRIS level in the HME cells (see also [20], [22], [23]), while high BRCA1-IRIS level in the IRIS cells (Figure 1E). Moreover, BRCA1-IRIS silencing abolished the signal in both cell lines (Figure 1E). 3) Immunohistochemical staining of invasive breast cancer samples with pre-absorbed antibody (i.e., pre-incubated with intron 11 peptide). This treatment led to complete absence of signal (Figure 1F and arrow in 1G). Thus, we concluded that we have developed a BRCA1-IRIS specific monoclonal antibody that can be used in immunohistochemistry on paraffin-embedded tumor samples. However, a relatively harsh antigen retrieval step consisting of incubating the sections for 20 minutes at 37°C with 10 µM pepsin is required.

To analyze BRCA1-IRIS expression in breast tumors, two cohorts of paraffin embedded tissue microarrays (TMA) were acquired. The first was a test cohort; a commercial TMA (Biomax. us) that consisted of 66 normal/cancer adjacent tissues, 180 ductal carcinoma *in situ* (DCIS), 100 invasive, and 165 metastatic breast tumor samples. The second was a confirmation cohort, consisting of disease-free adult tissues (including; kidney, liver, placenta, spleen and mammary tissues) and 326 breast tumor samples (different stages) acquired from the Hawaiian *Surveillance, Epidemiology and End Results* (SEER) collection. Both sets were constructed in quadruplicate, each containing one sample from a different region of a tumor at 4 µm.

Following immunohistochemical staining of the test cohort, BRCA1-IRIS-positive vs. negative cells was counted in at least 10 high power fields of each tumor. The data showed that BRCA1-IRIS is expressed at very low level in normal breast tissues (Figure 1H and arrow in 1I), at moderate level in DCIS tumors (Figure 1J and see BRCA1-positive [solid arrow] vs. -negative [dashed arrow] in Figure 1K), and at very high level in invasive (Figure 1L and arrow in 1M) and metastatic tumors (not shown). Taken together we concluded that BRCA1-IRIS expression increases in breast cancer as early as DCIS.

In keeping with our earlier observations that showed BRCA1-IRIS exclusive chromatin association [20], and its function in replication [22] and transcription [23] (processes known to occur at the nuclear matrix and in the nucleolus [27]–[30]). Here too BRCA1-IRIS staining was predominantly confined to the nuclear matrix (see arrow in Figure 1M) and the nucleolus (see arrowheads in Figure 1M).

### Immunohistochemical features of BRCA1-IRIS-positive breast tumors

To confirm the upregulation of BRCA1-IRIS in breast tumors, the second cohort was stained with the new anti-BRCA1-IRIS antibody. Disease-free liver (Figure S1A and S1B), placenta (Figure S1C and S1D), and spleen (Figure S1E and S1F, see also [20]) tissues all stained positive, whereas disease free kidney and normal mammary glands (see Figure 1H and I) were negative (Figure S1G and S1H). These data show that BRCA1-IRIS expression is also high in highly proliferative tissues.

We recently showed that BRCA1-IRIS overexpression promotes expression of AKT1, AKT2, p-AKT and survivin in human ovarian normal and cancer cell lines [25]. To evaluate whether this also occur in breast tumors *in vivo*, TMA slides from the same tumor blocks were immunohistochemically stained with BRCA1-IRIS, AKT1, AKT2, p-AKT, survivin and BRCA1/p220 specific antibodies. Staining was scored as follows; 0 = no staining (<1% of the cells stained); 1+ = weak (1–10% of the cells stained); 2+ = medium (10–50% of the cells stained); 3+ = strong (>50% of the cells stained), and staining scores <10% were considered negative tumors.

**Figure 2:**
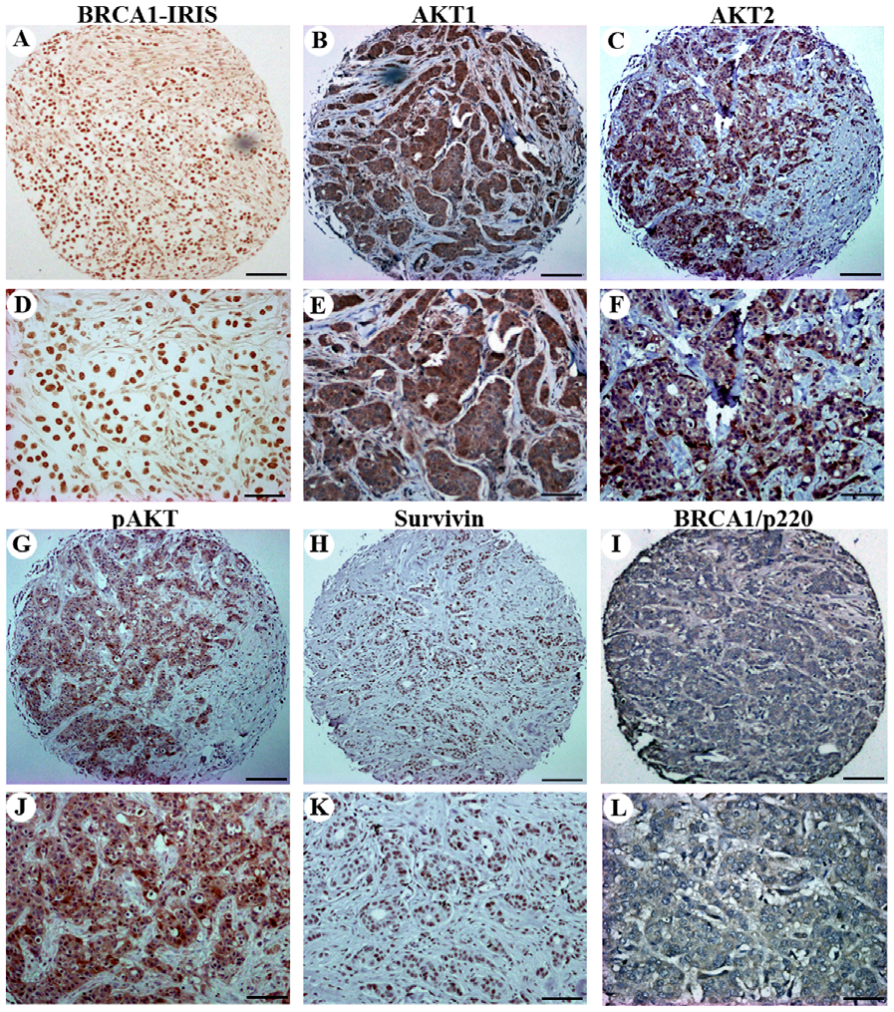
Expression of BRCA1-IRIS, AKT1, AKT2, p-AKT, survivin and BRCA/p220 in breast tumors. Representative sections of TN/BL breast tumor tissues showing low (A, B, C, G, H, and I) and high (D, E, F, J, K, and L) magnification images of sections stained for BRCA1-IRIS (A and D), AKT1 (B and E), AKT2 (C and F), p-AKT (D and J), survivin (H and K), and BRCA1/p220 (I and L). Bars are 400 µm in A–D and 200 µm in E–H.

In this conformational cohort as well, the majority, 84% (n = 274) of the tumors were BRCA1-IRIS-positive, while only 16% (n = 52) were BRCA1-IRIS-negative. The majority of the BRCA1-IRIS-positive tumors (Figure 2A and D) stained positive for AKT1, AKT2, p-AKT and survivin and negative for BRCA1/p220. Indeed 176 (64%, see example in Figure 2B and 2E), 180 (65%, see example in Figure 2C and 2F), 188 (68%, see example in Figure 2G and 2J) and 175 (63%, see example in Figure 2H and 2K) of the 274 BRCA1-IRIS-positive tumors stained positive for AKT1, AKT2, p-AKT and survivin, respectively, whereas only 4 (1%) of the BRCA1-IRIS-positive tumors stained positive for BRCA1/p220 (Figure 2I and 2L). Note that BRCA1/p220-negative tumors often show no or little cytoplasmic staining with BRCA1/p220 antibody [31]. In contrast, 19 (37%), 18 (35%), 15 (29%) and 17 (33%) of the BRCA1-IRIS-negative tumors (n = 52) stained positive for AKT1, AKT2, p-AKT and survivin, respectively, whereas the majority were BRCA1/p220-positive (data not shown).

**Figure 3:**
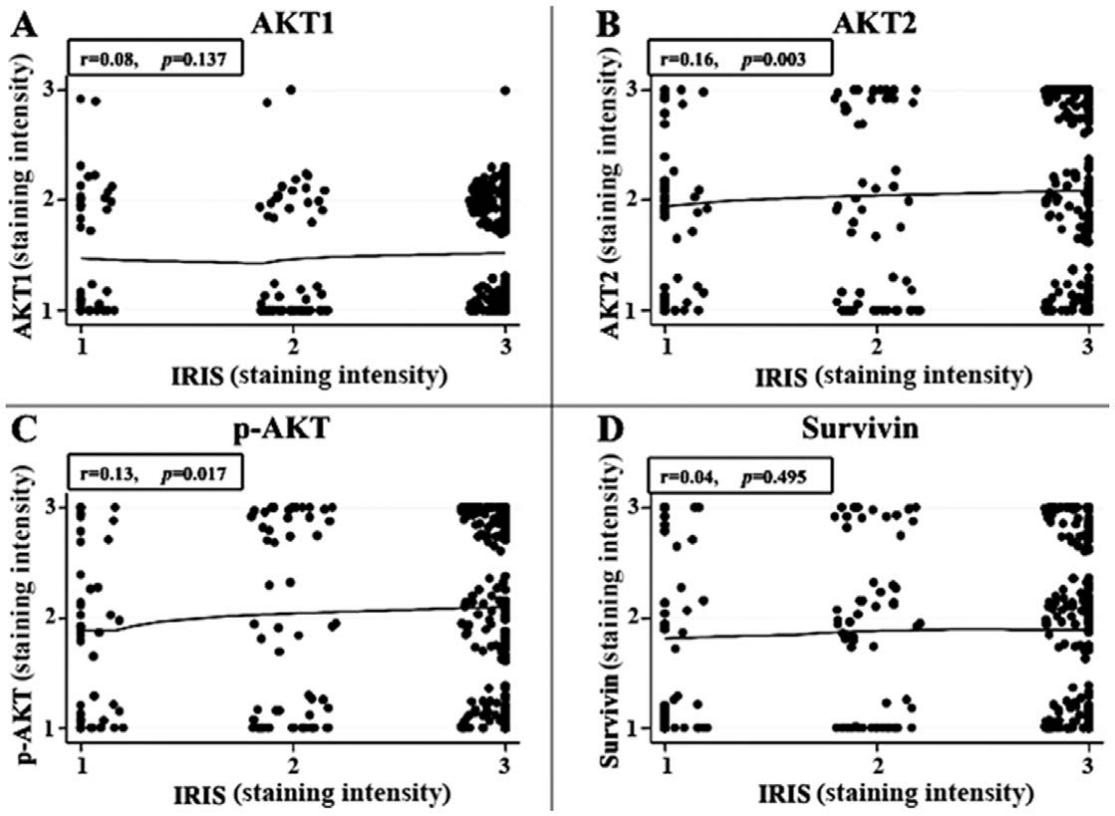
Correlations between BRCA1-IRIS expression and AKT1, AKT2, p-AKT and survivin in breast tumor samples. Nonparametric *Spearman rank correlation test* comparing BRCA-IRIS and AKT1, AKT2, p-AKT, and survivin on 326 breast tumors TMAs. The staining for each marker was scored as described in the text and the results were blotted. Insets show Spearman correlation coefficient (r) and *p*-value for each correlation.

We then examined the associations of expression-levels between BRCA1-IRIS and AKT1, AKT2, p-AKT, and survivin in BRCA1-IRIS-positive tumors using State v.11 to calculate Fisher's exact *p* values (Table 1) and Spearman correlation coefficients (r, Figure 3). According to the Fisher's exact test, significant associations between BRCA1-IRIS and AKT1 (*p-value* = 0.012, Table 1), or AKT2 (*p* = 0.006, Table 1) or p-AKT (*p* = 0.030, Table 1), but not survivin (*p* = 0.542, Table 1) were observed. Spearman correlation coefficient test, on the other hand, showed moderate yet significant correlations between the expression of BRCA1-IRIS and AKT2 (Spearman rank correlation r = 0.16, *p* = 0.003, Figure 3) or p-AKT (r = 0.13, *p* = 0.017, Figure 3) only. These data suggest that even in breast tumors BRCA1-IRIS overexpression correlates with high expression/activation of AKT (especially AKT2) and to a lesser extent survivin.

**Table 1:** The association between BRCA1-IRIS overexpression and overexpression/activation of AKT and survivin in breast tumors.

Staining Score	AKT1 (a p = 0.012)			AKT2 (p = 0.006)			p-AKT (p = 0.030)			Survivin (p = 0.54)			Total (%)
	0–1 (%)	2 (%)	3 (%)	0–1 (%)	2 (%)	3 (%)	0–1 (%)	2 (%)	3 (%)	0–1 (%)	2 (%)	3 (%)	
**0–1 (%)**	27 (55)	20 (41)	2 (4)	19 (39)	21 (43)	9 (18)	20 (40)	16 (33)	13 (27)	24 (48)	13 (27)	12 (25)	**49 (15)**
**IRIS 2 (%)**	37 (64)	19 (33)	2 (3)	25 (43)	13 (22)	20 (35)	27 (46)	9 (16)	22 (38)	20 (34)	23 (40)	15 (26)	**58 (18)**
**3 (%)**	104 (48)	112 (57)	1 (0)	61 (28)	65 (30)	91 (42)	65 (30)	59 (27)	93 (43)	86 (40)	69 (31)	62 (29)	**217 (67)**
**Total (%)**	**168 (52)**	**151 (47)**	**5 (1)**	**105 (32)**	**99 (31)**	**120 (37)**	**112 (34)**	**84 (26)**	**128 (40)**	**130 (40)**	**105 (31)**	**89 (27)**	**324 (100)**

a is Fisher's exact *p*-value.

### High expression BRCA1-IRIS in HER2^+^ and TN/BL breast tumors

HER2^+^ and TN/BL, as previously stated, are the most aggressive breast tumor subtypes. HER2^+^ tumors often acquire resistance to Herceptin (the only targeted therapy for these tumors), and TN/BL while responsive to chemotherapies, thus far, we have no targeted therapies for these tumors. To test whether BRCA1-IRIS is a useful chemotherapeutic target for either of these tumor subtypes, we identified a cohort of HER2^+^ (n = 32) and a cohort of TN/BL (n = 72) tumors. Stained tumors with BRCA1-IRIS, AKT (AKT1+AKT2) and survivin were then analyzed. There was also some information with regards to tumor grade and tumor stage available.

In the HER2^+^ cohort (n = 32), 26 tumors (81%) were BRCA1-IRIS-positive (Table 2), and only 6 tumors (19%) were BRCA1-IRIS-negative. There was significant strong correlation between BRCA1-IRIS expression and AKT (r = 0.752, *p* = 9×10^−6^, Table 2) or survivin (r = 0.859, *p* = 1×10^−6^, Table 2) expression. Six HER2^+^ tumors were BRCA1-IRIS-negative (19%, Table 2), and from these, 3 tumors (50%) were AKT-positive (*p* = 0.621, Table 2) and 5 tumors (83%) were survivin-positive (*p* = 0.822, Table 2). Further, in the TN/BL cohort (n = 72), 63 (88%) were BRCA1-IRIS-positive (Table 2), while only 9 (12%) were BRCA1-IRIS-negative. There was significant strong correlation between BRCA1-IRIS expression and AKT (r = 0.748, *p* = 0.00043, Table 2) or survivin (r = 0.834, *p* = 0.0038, Table 2) expression. Nine TN/BL tumors were BRCA1-IRIS-negative (12%, Table 2), and from these 7 tumors (78%) were AKT-positive (*p* = 0.492, Table 2) and 7 tumors (78%) were survivin-positive (*p* = 0.432, Table 2). Taken together, these data show that BRCA1-IRIS is overexpressed in two of the most aggressive breast tumor subtypes and that its overexpression correlates with increased AKT and survivin in these tumors.

**Table 2:** Relationships between BRCA1-IRIS level and marker expression and tumor characteristics in Her2^+^ and TN/BL breast cancer tumor samples.

	Her2^+^ (n = 32)		TN/BL (n = 72)	
	IRIS-positive	IRIS-negative	IRIS-positive	IRIS-negative
Characteristics	(%)	(%)	(%)	(%)
	(n = 26)	(n = 6)	(n = 63)	(n = 9)
**AKT-positive** (3+ and 2+)	19 (73)	3 (50)	54 (86)	7 (78)
**AKT-positive** (1+ and 0)	7 (27)	3 (50)	9 (14)	2 (22)
ar	0.752	0.002	0.748	0.009
b *p-value*	0.000009	0.621	0.00043	0.492
**Surv-positive** (3+ and 2+)	26 (100)	5 (83)	53 (84)	7 (78)
**Surv-positive** (1+ and 0)	0 (0)	1 (17)	10 (16)	2 (22)
ar	0.859	0.03	0.834	0.008
b *p-value*	0.000001	0.822	0.0038	0.432
**Tumor Grade (as modified nuclear grade)**				
**1**	0 (0)	0 (0)	0 (0)	0 (0)
**2**	12 (46)	6 (100)	25 (40)	9 (100)
**3**	14 (53)	0 (0)	38 (60)	0 (0)
c *p-value*	0.0221	0.0426	0.0152	0.667
**Tumor Stage**				
*In situ*	0 (0)	0 (0)	3 (5)	0 (0)
Localized	11 (44)	3 (50)	35 (56)	6 (67)
Lymph-node	8 (31)	3 (50)	20 (32)	3 (33)
Distant Mets	7 (25)	0 (0)	5 (8)	0 (0)
c *p-value*	0.0201	0.617	0.0184	0.0421

a Spearman rank coefficient test correlation (r),

b Spearman rank coefficient test *p*-value,

c To compare multiple groups with one control group, analysis of variance (ANOVA) was used.

*p*-values (two-sided) <0.05 were considered statistically significant.

### High BRCA1-IRIS in aggressive HER2^+^ and TN/BL tumors

All HER2^+^/BRCA1-IRIS-negative tumors (n = 6) were grade 2 (*p* = 0.0426, Table 2). One half (n = 3) of the HER2^+^/BRCA1-IRIS-negative tumors was localized tumors, while the other half (n = 3, *p* = 0.617) was lymph node positive tumors (Table 2). In contrast, 12 (46%) HER2^+^/BRCA1-IRIS-positive tumors were grade 2 and 14 (53%) were grade 3 tumors (*p* = 0.0221, Table 2). Eleven (44%) HER2^+^/BRCA1-IRIS-positive tumors were localized tumors, 8 (31%) were lymph-node positive tumors, and 7 (25%) showed distant metastases (*p* = 0.0201, Table 2). Further, all TN/BL/BRCA1-IRIS-negative tumors (n = 9) were grade 2 tumors (*p* = 0.667, Table 2). Six (67%) of the TN/BL/BRCA1-IRIS-negative tumors were localized tumors and 3 (33%) were lymph-node positive tumors (*p* = 0.0421, Table 2). In contrast, 25 (40%) TN/BL/BRCA1-IRIS-poitive tumors were grade 2 tumors; 38 (60%) were grade 3 tumors (*p* = 0.0152). Thirty-five TN/BL/BRCA1-IRIS-positive tumors (56%) were localized tumors; 20 (32%) were lymph-node positive tumors; and 5 (8%) showed distant metastasis (*p* = 0.0184, Table 2).

Finally, BRCA1-IRIS positivity significantly reduced age at diagnosis in HER2^+^ (51.4±16.2 vs. 64.5±13.2, *p≤0.05*, n = 14) and TN/BL (48.9±10.1 vs. 70.8±6.8, *p≤0.01*, n = 23) tumors. Surprisingly, however, TN/BL/BRCA1-IRIS-positive tumor size was significantly larger than that of HER2^+^/BRCA1-IRIS-positive tumors (30.6±19.3 vs. 16.1±9.7, *p≤0.05*). Taken together, these data show that the increase in BRCA1-IRIS expression correlates with enhanced aggressive breast tumor behavior and adverse outcomes, especially in patients with HER2^+^ or TN/BL breast tumor subtypes.

**BRCA1-IRIS overexpressing HME cells form subcutaneous tumors in SCID mice** To directly assess BRCA1-IRIS tumor inducing potential, a xenograft mouse model was developed. TERT-immortalized HME cells (hereafter HME/TERT) overexpressing LT, BRCA1-IRIS, Ras^V12^ (negative controls, see [26]), LT and Ras^V12^ (positive control, see [26]) or LT and BRCA1-IRIS were subcutaneously injected in 6 to 8 week-old female SCID mice (n = 10/cell line). All cell lines expressed luciferase to be visualized in living animals and tumor formation was analyzed by Xenogen *in vivo* imaging weekly or by caliper measurement every 3^rd^ day. According to published data, high-level Ras^V12^ is required to induce tumor formation using immortalized HME cells [26]. Indeed, the HME cells we used expressed equally high levels of LT, Ras^V12^ and BRCA1-IRIS (Figure S2A).

**Figure 4:**
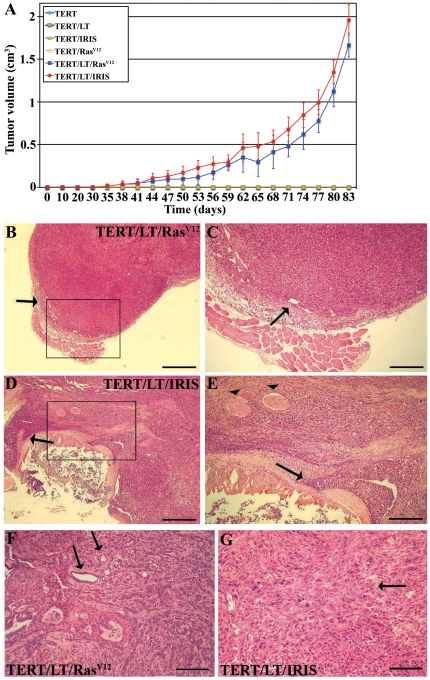
BRCA1-IRIS-induced tumors show more aggressive phenotypes than Ras^V12^-induced tumors. (A) The volume of subcutaneous tumor formed in SCID mice injected with immortalized HME cells expressing, LT, Ras^V12^, BRCA1-IRIS alone, or LT and Ras^V12^, or LT and BRCA1-IRIS. Results are expressed as the mean of 6 tumors ± SD at the indicated time points after injection. Histology of these subcutaneous tumors as shown under light microscope of H&E stained sections. (B–C) Show low and high magnification images, respectively of a poorly differentiated Ras^V12^-induced tumor. (D–E) Show low and high magnification images, respectively of a poorly differentiated BRCA1-IRIS-induced tumor. Only in BRCA1-IRIS-induced tumors, we were able to see tumors cells invading mouse muscle tissues (arrow in E) or nerves (arrowheads in E). (F) Show poorly differentiated Ras^V12^-induced tumor with large pleomorphic nuclei and prominent nucleoli as well as ductal-like structures (arrows in F). (G) Show poorly differentiated BRCA1-IRIS-induced tumor with large pleomorphic nuclei and prominent nucleoli and a large component of spindle-like tumor cells (see arrow in G). Bars, 800 µm in B and D, 400 µm in C, and E, 200 µm in F and G.

As expected, immortalized HME cells overexpressing 1 oncogene (LT, Ras^V12^ or BRCA1-IRIS) failed to form tumors (Figure 4A and Figure S2B), whereas 8 of 10 mice injected with immortalized HME cells expressing LT/Ras^V12^ developed tumors (see blue line in Figure 4A and Figure S2B). More importantly, 9 of 10 mice injected with immortalized HME cells expressing LT/BRCA1-IRIS also developed tumors (see red line in Figure 4A and Figure S2B). BRCA1-IRIS-induced tumors were observed earlier than Ras^V12^-induced tumors (45 vs. 53 days), although the difference was not significant (*p* = 0.09, compare red to blue line in Figure 4A). Both tumors took ∼12 weeks to reach ∼1.5 cm^3^ (the allowable size, see blue and red lines in Figure 4A).

### Histological differences between BRCA1-IRIS- and Ras^V12^-induced tumors

BRCA1-IRIS- and Ras^V12^-induced subcutaneous tumors were embedded in paraffin, sectioned at 4 µm in the middle of each tumor, stained with hematoxylin and eosin (H&E) and blindly analyzed by 2 pathologists. BRCA1-IRIS-, as well as Ras^V12^-induced tumors were poorly differentiated carcinomas with histological features of high-grade epithelial malignancies, contained areas with glandular differentiation (Figure 4B–E) and showed areas with conspicuous squamous cell differentiation with prominent keratinization (data not shown). Although a spindle cell component (occasionally observed in human high-grade breast cancers) was occasionally observed in both tumors, this component was more prominent in BRCA1-IRIS-induced tumors (see arrow in Figure 4G). In contrast, Ras^V12^- and not BRCA1-IRIS-induced tumors showed areas with ductal-like structures (see arrows in Figure 4F). These data suggest that BRCA1-IRIS overexpression promotes formation of more aggressive tumors.

In keeping with that Ras^V12^-induced tumors were well circumscribed (see arrows in Figure 4B and 4D), whereas BRCA1-IRIS-induced tumors showed destructive growth patterns (see arrow in Figure 4C and 4E) manifested by invasion of the adjacent native mouse parenchyma and surrounding tissues, such as bone (not shown) and neural tissues (see arrowheads in Figure 4E). Finally, although BRCA1-IRIS-induced tumors were slightly larger in size than Ras^V12^-induced tumors, the difference was not significant (*p* = 0.09, Figure 4A). It is possible that because BRCA1-IRIS-induced tumors were highly proliferative (see higher mitotic figures per high-power field, Table 3) and at the same time highly necrotic than Ras^V12^-induced tumors (for comparison throughout the tumors see Figure S3), BRCA1-IRIS-induced tumors perhaps appear smaller. These data show that BRCA1-IRIS overexpression is oncogenic promoting subcutaneous tumor formation in SCID mice.

**Table 3:** Immunohistochemical and pathologic characteristics significantly different in LT/Ras^V12^ *vs.* LT/IRIS subcutaneous tumors.

	LT/Ras^V12^ (n = 8)	LT/IRIS (n = 9)	*p*-value
	N (%)	N (%)	
**BRCA1-IRIS/20 high power fields**			
Total	120	116	
Nuclear	120 (100)	116 (100)	
Intensity	0 (0)	0 (0)	
**BRCA1/p220/20 high power fields**			
Total	99	97	
Nuclear	83 (84)	10 (11)	≤0.01
Intensity	16 (16)	87 (89)	≤0.01
**AKT1/20 high power fields**			
Total	104	108	
Positive	46 (44)	108 (100)	≤0.05
Intensity	2 (40)	5 (100)	≤0.01
**AKT2/20 high power fields**			
Total	103	106	
Positive	61 (58)	106 (100)	≤0.05
Intensity	2 (40)	5 (100)	≤0.01
**p-AKT/20 high power fields**			
Total	101	102	
Positive	59 (59)	102 (100)	≤0.05
Intensity	2 (40)	5 (100)	≤0.01
**Survivin/20 high power fields**			
Total	102	107	
Positive	24 (24)	57 (53)	≤0.05
Intensity	2 (40)	5 (100)	≤0.05
**Mitotic figures/20 high power fields**			
Total	102	95	
positive	4 (4)	14 (15)	≤0.01

### Immunohistochemical differences between BRCA1-IRIS- and Ras^V12^-induced tumors

**Figure 5:**
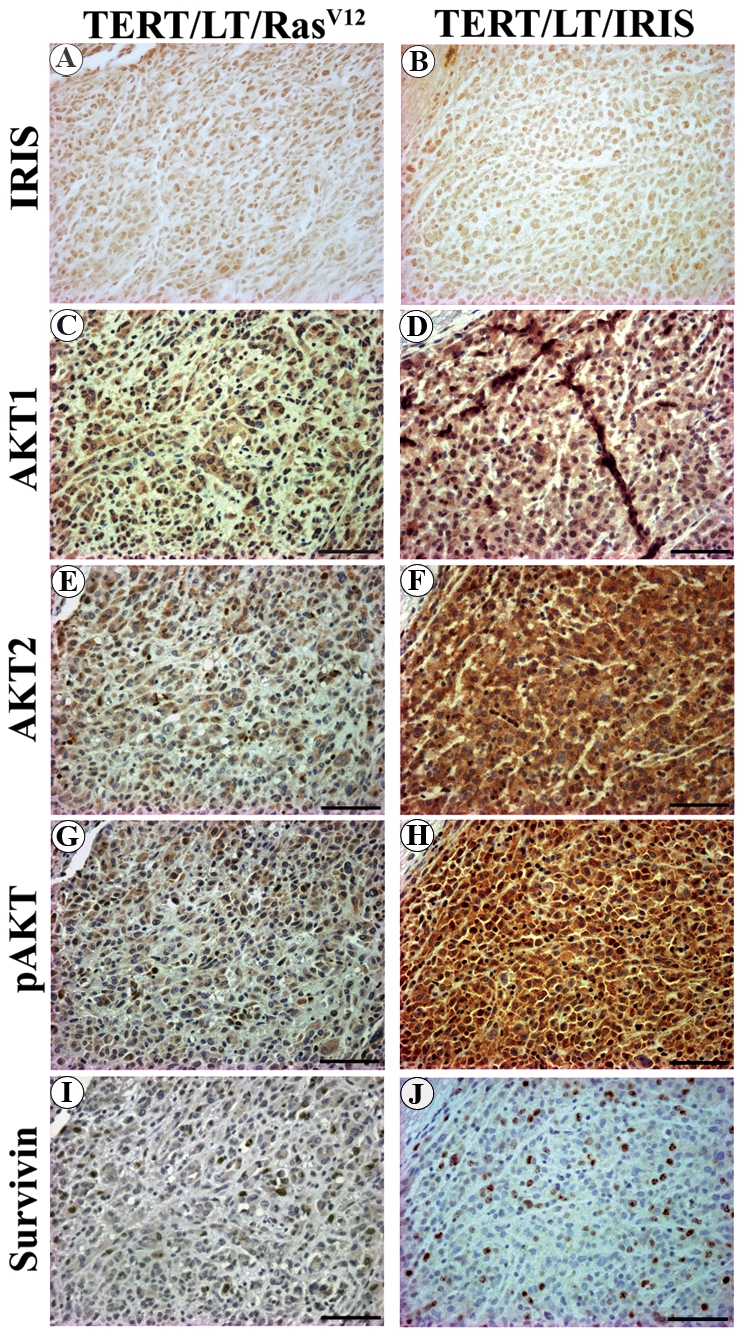
Immunohistochemical analysis of Ras^V12^ or BRCA1-IRIS-induced subcutaneous tumors. Representative high magnification sections from tumor xenografts developed in mice injected with HME cells expression TERT/LT/Ras^V12^ (A, C, E, G and I) or TERT/LT/IRIS (B, D, F, H and J) stained for BRCA1-IRIS (A and B), AKT1 (C and D), AKT2 (E and F), p-AKT (G and H) and survivin (I and J). Bar is 200 µm.

To extend these data to expression of the survival factors AKT1, AKT2, p-AKT, and survivin, adjacent sections from each tumor were immunohistochemically stained with BRCA1-IRIS, AKT1, AKT2, p-AKT, survivin, and BRCA1/p220 antibodies. Unexpectedly, we observed increase BRCA1-IRIS expression not only as would be expected in BRCA1-IRIS-induced tumors (Figure 5B) but also in Ras^V12^-induced tumors (Figure 5A). These data suggest that BRCA1-IRIS expression increases upon tumor initiation. Moreover, higher AKT1, AKT2, p-AKT, and survivin were detected in BRCA1-IRIS-induced tumors (see examples in Figure 5D, F, H, and J) compared to Ras^V12^-induced tumors (see examples in Figure 5C, E, G, and I).

Quantitative analysis showed that AKT1, AKT2, and p-AKT antibodies stained all the cells, while survivin antibody stained ∼50% of the cells per high power field in BRCA1-IRIS induced tumors (Table 3). In contrast, in Ras^V12^-induced tumors, the AKT1, AKT2, and p-AKT antibodies stained ∼50% of the cells, and survivin antibody stained ∼25% of the cells per high power field (Table 3). The staining intensity per cell for each of these markers was much higher in BRCA1-IRIS-induced tumors when compared to cells from Ras^V12^-induced tumors (Table 3). These data show that, like cultured HOSE cells [23], BRCA1-IRIS overexpression triggers the expression and activation of AKT and survivin in HME cells, *in vivo*.

Interestingly, Ras^V12^-induced tumors maintained high BRCA1/p220 expression (Figure S4a, c and e and Table 3), whereas in BRCA1-IRIS-induced tumors, the expression of BRCA1/p220 was virtually absent (Figure S4b, d and f and Table 3). Another prominent difference is that Ras^V12^-induced tumors stained positive for the epithelial markers; p63 and CK19, while BRCA1-IRIS-induced tumors stained negative (compare A to B in Figure S5). In contrast, BRCA1-IRIS-induced tumors stained positive for the mesenchymal marker; vimentin, whereas Ras^V12^-induced tumors stained negative (compare C to D in Figure S5). These data show that BRCA1-IRIS overexpression promotes formation of aggressive tumors, by upregulating expression of survival factors; such as AKT and survivin, suppressing expression of tumor suppressors; such as BRCA1/p220, and/or by inducing epithelial to mesenchymal transition (EMT), *in vivo*.

### Upregulation of rat BRCA1-IRIS in primary as well as invasive rat mammary tumors

It is difficult to study human breast cancer progression from an *in situ* tumor to invasive cancer. Rats treated with N-methyl-N-nitrosourea (NMU) develop primary *in situ* breast tumors that are similar to low-grade estrogen-receptor positive human breast cancers [32]. Serial transplantation of these primary tumors in syngeneic hosts leads to gradual progression to a higher-grade invasive disease [33]. Invasive tumors, as with those in humans, usually lose p63 expression and basement membrane integrity [33] and demonstrate a more mesenchymal phenotype with increased vimentin expression and decreased epithelial marker expression [33].

**Figure 6:**
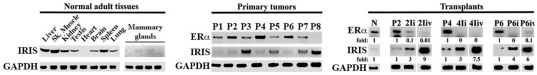
BRCA1-IRIS mRNA expression in NMU-induced primary and invasive breast tumors. (A) RT/PCR analysis of rat *BRCA1-IRIS* mRNA expression in normal adult rat tissues (left) and several adult rat mammary glands (right). (B) RT/PCR analysis of the expression of *ERα* and *BRCA1-IRIS* mRNAs in several primary rat breast tumors generated flowing NMU treatment (P1–P8). (C) RT/PCR analysis of the expression of *ERα* and *BRCA1-IRIS* mRNAs in normal gland (N), three primary tumors P2, P4 and P6 and the first (2Ii, 4Ii and 6Ii) or the fourth (2Iiv, 4Iiv and 6Iiv) invasive tumors generated following serial transplantation of the mentioned primary tumors.

To directly assess the involvement of BRCA1-IRIS in breast cancer progression, primary tumors from NMU-treated rats, as well as their invasive transplants, were analyzed for the expression of rat *BRCA1-IRIS* mRNA using RT/PCR (a rat BRCA1-IRIS specific antibody is currently unavailable). The primers used for this study detect a segment in the intron 11 of rat *BRCA1-IRIS* transcript. First, we analyzed the expression of rat *BRCA1-IRIS* mRNA in several rat adult tissues, including several normal mammary glands. Rat *BRCA1-IRIS* mRNA is expressed at high level in some tissues, e.g., spleen (Figure 6A), while at low to undetectable level in others, e.g., heart and normal mammary gland (Figure 6A). Rat *BRCA1-IRIS* mRNA expression varied in 8 primary tumors. Some primary tumors (e.g., P3, 5, 7 and 8, Figure 6B) expressed high levels, whereas others (e.g., P1, 2, 4 and 6 in Figure 6B) expressed low to undetectable levels. Interestingly, an inverse correlation with rat *ERα* mRNA expression in these primary tumors was observed (Figure 6B, see also [22]). These data suggest that rat *BRCA1-IRIS* mRNA increases in a subset of primary breast tumors that express low *ERα* mRNA (perhaps belonging to HER2^+^ or TN/BL tumor subtypes).

Next, 3 of the low rat *BRCA1-IRIS* mRNA expressing primary tumors, namely P2, 4 and 6 were transplanted for several rounds in syngeneic hosts. The expression of rat *BRCA1-IRIS* and *ERα* mRNAs were followed in these transplants using RT/PCR. *BRCA1-IRIS* mRNA rose 3–4 fold in these tumors during the 1^st^ transplants (compare 2Ii to P2, 4Ii to P4 and 6Ii to P6 in Figure 6C) and rose even further to 6–9 fold in the 4^th^ transplants (see 2Iiv, 4Iiv and 6Iiv in Figure 6C). Importantly, while these tumors expressed high *ERα* mRNA level as primaries (see P2, 4 and 6 in Figure 6C), in the transplants, *ERα* mRNA levels decreased significantly when rat *BRCA1-IRIS* mRNA levels increased (Figure 6C). These data show a gradual and continuous increase in rat *BRCA1-IRIS* mRNA expression during breast cancer progression/invasion, which correlates with significant decrease in the expression of rat *ERα* mRNA and strongly support the notion that BRCA1-IRIS overexpression is involved in breast cancer progression, most likely of the HER2^+^ and/or TN/BL subtypes.

## Discussion

Several lines of evidence presented in this study indicate that BRCA1-IRIS may act as a breast cancer oncogene that induces aggressive breast cancer when overexpressed. First, BRCA1-IRIS is overexpressed in the majority of tumors analyzed, especially from the HER2^+^ and TN/BL subtypes. Second, positive correlation between BRCA1-IRIS expression and the expression of the tumor inducing proteins, AKT and survivin, while negative correlation with the expression level of the tumor suppressor BRCA1/p220, especially in HER2^+^ and TN/BL tumors was detected as well. Third, BRCA1-IRIS overexpression was associated with poor prognosis and outcome in breast carcinoma of the HER2^+^ and TN/BL subtypes. Forth, BRCA1-IRIS overexpression transforms HME cells *in vitro* [22]–[25] and HME cells overexpressing BRCA1-IRIS (in combination with TERT and LT) form tumors *in vivo* (this study). Finally, breast tumor formation and progression in rats induced by the carcinogen, NMU was associated with induction in rat BRCA1-IRIS (mRNA) expression.

Although HME cells overexpressing TERT/LT/Ras^V12^ or /BRCA1-IRIS both developed subcutaneous tumors when injected in SCID mice (Figure 4), and although Ras^V12^-induced tumors also showed high BRCA1-IRIS expression (compare A to B in Figure 5 and c to d in Figure S4), only BRCA1-IRIS-induced tumors were invasive. This may be explained by the fact that only BRCA1-IRIS-induced tumors have lost expression of the tumor suppressor BRCA1/p220 (compare f to e in Figure S4). It is possible that loss of BRCA/p220 combined with BRCA1-IRIS overexpression generates much more aggressive, invasive, and/or metastatic tumors. Not surprisingly, there have been no Ras activating mutations ever detected in breast cancers. Alternatively, it is possible that the dramatic increase in AKT and survivin expression and/or activation in BRCA1-IRIS-induced tumors and not Ras^V12^-induced tumors (see Figure 5) generate tumor cells that are more aggressive. In fact, in cultured cells, BRCA1-IRIS, and not Ras^V12^ overexpressing cells showed high level of proliferation and low level of apoptosis (data not shown).

What is the mechanism responsible for enhanced BRCA1-IRIS expression in Ras^V12^-induced tumors? It was recently shown that oncogenic Ras overexpression decrease the expression of the mRNA 3′-UTR binding and destabilizing proteins, AUF1, during mammary cell transformation [34]. Interestingly, we recently found that the 3′-UTR of *BRCA1-IRIS* mRNA contained binding sites for AUF1 (submitted). It is possible that the downregulation of these factors by Ras^V12^ overexpression stabilizes BRCA1-IRIS mRNA, leading to BRCA1-IRIS protein overexpression. Another possibility is that oncogenic Ras affects BRCA1-IRIS transcription by up- and/or down-regulating specific transcription factors. In this regard it was shown recently that Ras^V12^ overexpression down-regulates the expression of vitamin D during mammary epithelial cell transformation [35]. Whether this affects BRCA1-IRIS transcription remains to be seen.

Although relatively similar size subcutaneous tumors were induced by Ras^V12^- or BRCA1-IRIS-overexpression in SCID mice (Figure 4A), only BRCA1-IRIS-induced tumors showed increased aggressiveness (Figure 4). It is possible that because Ras^V12^-induced tumors maintained high expression levels of the tumor suppressor BRCA1/p220, this then contributed to the low aggressive phenotype in these tumors as compared to BRCA1-IRIS-induced tumors that lost BRCA1/p220 expression. Alternatively, BRCA1-IRIS-idnuced tumors were more proliferative (Table 3), as well as more necrotic (Figure 4B), than Ras^V12^-induced tumors. This might contribute to the apparent smaller size they show.

Necrosis is a hallmark of increased hypoxia within the tumor, due to lack of adequate vascular supply [36]. Necrosis is known to increase chronic ischemia, leading to infarction that triggers both microphage infiltration in tumors and angiogenesis [36]. The fact that BRCA1-IRIS- and not Ras^V12^-induced tumors showed increased necrosis throughout the tumors (compare b, d and f to a, c and e in Figure S3), suggests that they are hypoxic and support the fact that they are much more invasive. It is also important to point out that necrosis was shown recently to be a typical representative of basal-like breast cancer [37], and that in breast cancer patients whose tumors overexpress HER2, higher levels of activated AKT2 and the hypoxic-induced protein; HIF-1α [38]–[40] were observed. AKT2 and HIF-1α activate transcription of a plethora of genes involved in cancer cells proliferation, survival, and progression [39], [40]. Thus, the fact that BRCA1-IRIS expression was high in these tumors is in line with them being aggressive (see above). Furthermore, recent evidence showed that Twist upregulates AKT2 expression in breast cancer cells leading to tumor development and progression [41]. Twist's role in the induction of the EMT is well known. In keeping with that BRCA1-IRIS- and not Ras^V12^-induced subcutaneous tumors showed increased expression of mesenchymal proteins, but decreased expression of epithelial proteins (see Figure S5).

In conclusion, we have delineated a novel BRCA1-IRIS-dependent oncogenic pathway through activation of AKT and survivin. Collectively, our data suggest that BRCA1-IRIS overexpression is associated with tumors that are often drug resistant ([24], [25] and this study), such as HER2^+^, TN/BL, and perhaps BRCA1/p220-associated tumors. Finally, our data provide proof of concept that chemotherapeutic targeting of BRCA1-IRIS might decrease the drug resistance phenotype of these tumors and their metastatic potential.

## Materials and Methods

### Cell culture and establishment of doxycycline-dependent BRCA1-IRIS expressing HME cell lines

Human mammary epithelial (HME) cells were cultured in MEGM modified minimum medium (Lonza). BRCA1-IRIS full-length cDNA including the entire 3′-UTR was amplified using PCR from HME total RNA using primers described earlier [20] and was cloned into the pRevTRE plasmid (Clontech). The authenticity of the construct was verified by sequencing. HME cells transfected with the pTet-ON vector (the inducer, Clontech) were infected with retrovirus pRev-TRE-BRCA1-IRIS+3′UTR, selected with 150 µg/ml hygromycin B (Sigma), and clones were identified. Doxycycline (1–2 µg, Clontech) inducible expression of BRCA1-IRIS was monitored by real time RT/PCR and/or Western analysis using His- and/or BRCA1-IRIS-specific antibody (see Figure 1E).

### Antibodies

Anti-human BRCA1-IRIS antibody was raised in mice immunized with the BRCA1-IRIS intron-11 peptide (GIGTRFLCLPQSIYRSELNVYAFGEHILQISKYS) fused to glutathione *S*-transferase (GST) synthesized in *Escherichia coli* and purified on glutathione Sepharose-4B (GSSH) beads as described earlier [20]. For immunohistochemistry staining, this antibody was used at 1∶50 dilution as a hybridoma culture medium. The specificity of this antibody was first confirmed by depletion on a column containing GST-intron 11 peptide, followed by Western analysis and immunohistochemistry analysis, and in both cases no signal was detected. Rabbit anti-AKT1 (C73H10, produced against a synthetic peptide surrounding Leu110 of human AKT1 protein), and -AKT2 (D6G4, produced by immunizing animals with a synthetic peptide corresponding to residues in human AKT2) monoclonal antibodies, were from Cell Signaling (Beverly, MA) used at 1∶400 dilutions for immunohistochemistry staining, and did not cross-react. Rabbit anti-p-AKT antibody was a mixture (1∶1, i.e. 1∶50 dilution) of anti-phospho-AKT (Ser473, 736E11, produced by immunizing animals with a synthetic phospho-peptide corresponding to residues surrounding Ser473 of AKT1) and -phospho-AKT (Thr308, C31E5E, produced by immunizing animals with a synthetic phospho-peptide corresponding to residues around Thr308 of AKT1), and were from Cell Signaling. Both detect endogenous levels of AKT1 only when phosphorylated at Ser473 or Thr308, and detect AKT2 and AKT3 only when phosphorylated at equivalent sites. Rabbit polyclonal anti-survivin antibody (abcam, ab469) was used. Mouse monoclonal anti-BRCA1/p220 “SG11” (detects an epitope in the last 17 amino acid of BRCA1/p220) was used to detect BRCA1/p220 and 9E10 was used to detect Myc tag.

### Subcutaneous tumorigenicity assay and *in vivo* imaging of subcutaneous tumors

All animal experiments were approved by the University of Hawaii “Institutional Animal Care and Use Committee” (IACUC). The animals used were 6 to 8-week-old immune-compromised athymic SCID (NOD.CB17-*Prkdc* ^scid^/J, Jackson Laboratory) female mice. The Weinberg laboratory [26] recently showed that HME cells form xenograft or orthotopic tumors in SCID mice only when expressing 3 oncogenes (TERT, LT and X [variable]). Thus, to study the oncogenic effect of BRCA1-IRIS, we subcutaneously injected 5 million HME cells expressing TERT, TERT/LT, TERT/BRCA1-IRIS, or TERT/Ras^V12^ (as negative controls), TERT/LT/Ras^V12^ (as positive control), and compared those to cells expressing TERT/LT/BRCA1-IRIS. All cell lines also expressed luciferase and were injected in 200 µl of PBS/matrigel (1∶1) using a 25-gauge needle.

Tumors were monitored with the IVIS luciferase machine (Xenogen®) weekly. In brief, mice were injected intraperitoneally using 25G needle with 100 µl of D-luciferin solution (Xenogen®) prepared at 15 mg/ml in PBS, and anesthetized using a mix of oxygen and isoflurane gas. Tumors were visualized by a CCD camera within 15 minutes; during which mice were maintained asleep by placing them right (injection) side up and their noses in a nose-cone with a flow of anesthesia gas. Pictures of the tumors are shown in supplementary information. Tumor size was measured every third day by caliper (Life Sciences Instruments). Tumor initiation was defined as the time when tumors were 3 mm in diameter. Tumors typically have a timeframe of 12 weeks from the time of cell injection. Mice were sacrificed when the tumors grew to >1.5 cm in diameter or after 12 weeks of monitoring. Tumor volume was calculated with the formula 4/3πr^3^ (where r is the tumor radius). Tumors were fixed in formalin immediately following dissection and cut at 4 µm for histological and immunohistochemical analysis.

### Immunohistochemical analysis of paraffin-embedded tumor samples

Mouse tumors were fixed in formalin immediately following resection for 24 hours, PBS for another 24 hours (if the time fell over the weekend); otherwise, they were directly embedded in paraffin. For immunohistochemical staining, human or mouse tumor sections were deparaffinized and rehydrated with deionized water. Epitope retrieval for AKT1, AKT2, p-AKT, survivin and BRCA1/p220 was done by heating the slides in 10 µM citrate buffer (pH 6.0) using an electric pressure cooker, ∼15 *psi* at 95–120°C for 40 minutes, then rinsed in wash buffer for 5–20 minutes prior to staining. Epitope retrieval for BRCA1-IRIS staining was done by incubating the slides for 20 minutes at 37°C with 10 µM pepsin. On an automated system (Dako Autostainer) slides were exposed to 3% hydrogen peroxide for 5 minutes, followed by incubation with 1° antibody for 30 minutes, followed by several washes with Tris-buffered saline (TBS), and then incubation with 2° antibody for 30 minutes using the HRP polymer system. Slides were then developed with 3,3′-diaminobenzidine (DAB) for 5 minutes, counterstained with Meyer's hematoxylin for 5 minutes, and cover-slipped. In some experiments, a rabbit polyclonal BRCA1-IRIS antibody (also generated against an epitope in intron11 part of BRCA1-IRIS) was used and gave us identical results.

### Scoring for immunohistochemical staining of the slides

Here, we used two cohorts of breast tumor samples one set was purchased from Biomax.us, that contained 66 normal/cancer adjacent samples, 180 DCIS, 100 invasive tumors and 165 metastatic tumor samples. The second cohort was obtained from archived retrospectively collected tumors by the *Hawaiian Surveillance, Epidemiology and End Results* (SEER) Program, that contained in addition to 326 breast tumor samples, several disease-free tissue samples (i.e., kidney, liver, placenta, spleen, and normal breast) that were used as positive controls. All work with human tumor samples was approved by the University of Hawaii IRB committee and was performed after obtaining permission to use the archived samples, and all data was either anonymized or made anonymous by the researchers. As negative control, BRCA1-IRIS and the other 1° antibodies were replaced with TBS or by antibodies depleted prior to incubation with sections. Expression of membranous, cytoplasmic, or nuclear staining for the different antibodies was scored as follows; 0 = negative if <1% of the cells stained; 1+ = weak if between 1–10% of cells stained; 2+ = medium if between 10%–50% of cells stained; 3+ = strong if >50% of cells stained. Scores of 0 and 1+ were combined as negative tumors. All tumors and staining were evaluated under 4× and 10× magnifications, blindly, by 2 pathologists. The same criteria were used for the mouse tumors.

### Statistical analysis

Comparisons of statistical differences were done using the Student *t*-test for paired and unpaired data. Statistical significance was assumed at a *p*-value of ≤0.05. To compare multiple groups with 1 control group, analysis of variance (ANOVA) was used. The criterion for significance (*p*-value) was set as mentioned in the figures. The correlation between the level of BRCA1-IRIS expression and the histological grade was analyzed using the Fisher exact test. Nonparametric correlation test *(Spearman rank correlation test)* using Spearman correlation coefficient (r) was done between expressions of BRCA1-IRIS and AKT1, AKT2, p-AKT, and survivin.

## Supporting Information

Figure S1 **Expression of BRCA1-IRIS in unaffected patient's tissues.** Tissue samples from liver (A and B), placenta (C and D), spleen (E and F), and kidney (G and H) were all stained with mouse anti-BRCA1-IRIS monoclonal antibody. Note lack of BRCA1-IRIS expression in adult kidney tissue, while high expression in adult liver, placenta, and spleen tissues.
(TIF)

Figure S2 **Subcutaneous tumor formation using HME cells expressing TERT/LT/Ras^V12^ or /BRCA1-IRIS.** (A) Representative images of luciferase signals in mice injected with HME/Luc cells expressing TERT, TERT/LT, TERT/IRIS, TERT/LT/Ras^V12^, or TERT/LT/IRIS mixed with matrigel at day 1, week 5 or week 10 following cell injection.
(TIF)

Figure S3 **Pronounced necrosis in BRCA1-IRIS-induced and not Ras^V12^-induced tumors.** (A, C and E) are sections at different levels; top (A), middle (C) and bottom (E) of a Ras^V12^-induced tumor. (B, D and F) are sections at different levels; top (B), middle (D) and bottom (E) of BRCA1-IRIS induced tumor. Note the pronounced necrosis at all levels in BRCA1-IRIS- (arrows in B, D and F) and not Ras^V12^-induced tumors.
(TIF)

Figure S4 **Loss of BRCA1/p220 expression in BRCA1-IRIS- and not Ras^V12^-induced tumors.** Representative sections from Ras^V12^- (a, c, and e) or BRCA1-IRIS- (b, d, and f) induced tumors stained with H&E (a and b), for BRCA1-IRIS (c and d) or BRCA1/p220 (e and f).
(TIF)

Figure S5 **Loss of epithelial marker and gain of mesenchymal marker expression only in BRCA1-IRIS-induced tumors.** Representative sections from Ras^V12^- (A and C) or BRCA1-IRIS- (B and D) induced tumors double stained for p63 and cytokeratin (CK) 19 (A and B) or cyclin (Cyc) D1, and vimentin (C and D).
(TIF)
