# Supplementary figures and images for: BRCA1-IRIS Overexpression Promotes Formation of Aggressive Breast Cancers
Source: PLoS One. 2012 Apr 12;7(4):e34102. doi: 10.1371/journal.pone.0034102 (PMC3325250; doi:10.1371/journal.pone.0034102)

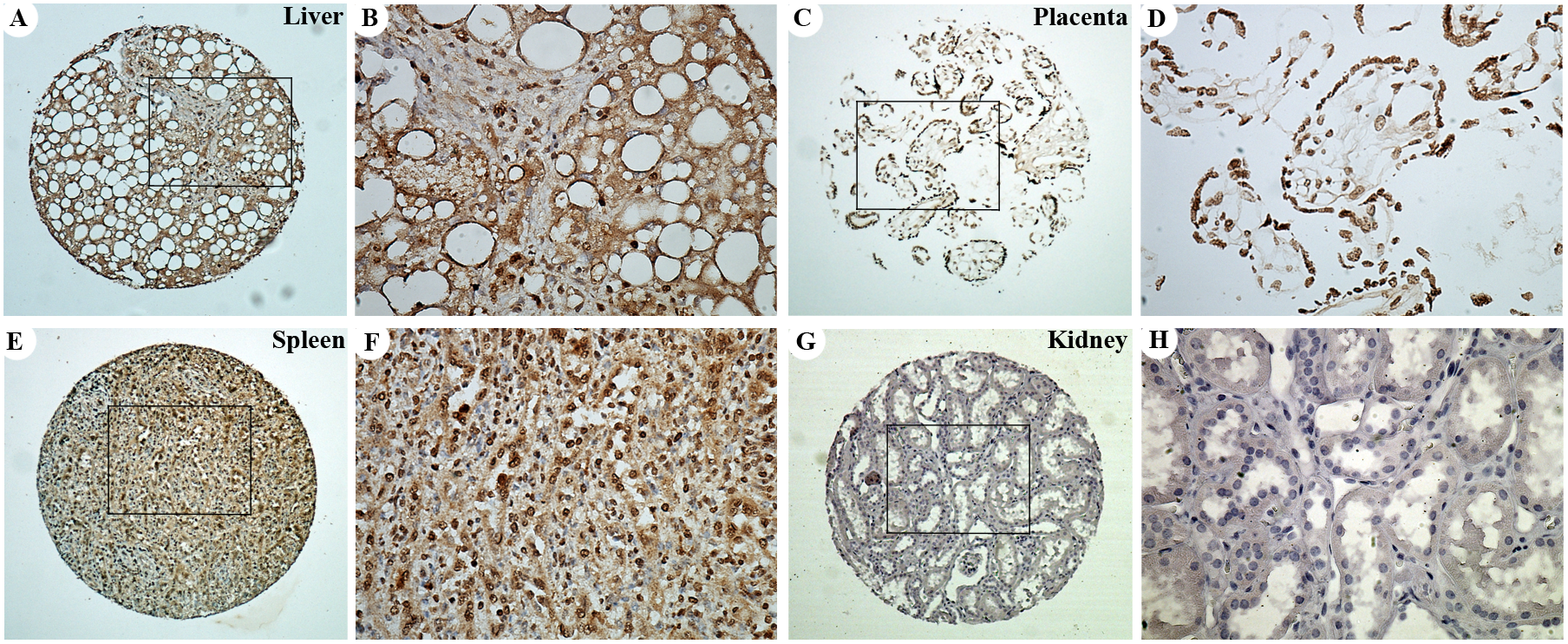

Supplement: Figure S1 — Expression of BRCA1-IRIS in unaffected patient's tissues. Tissue samples from liver (A and B), placenta (C and D), spleen (E and F), and kidney (G and H) were all stained with mouse anti-BRCA1-IRIS monoclonal antibody. Note lack of BRCA1-IRIS expression in adult kidney tissue, while high expression in adult liver, placenta, and spleen tissues. (TIF) [file pone.0034102.s001.tif]

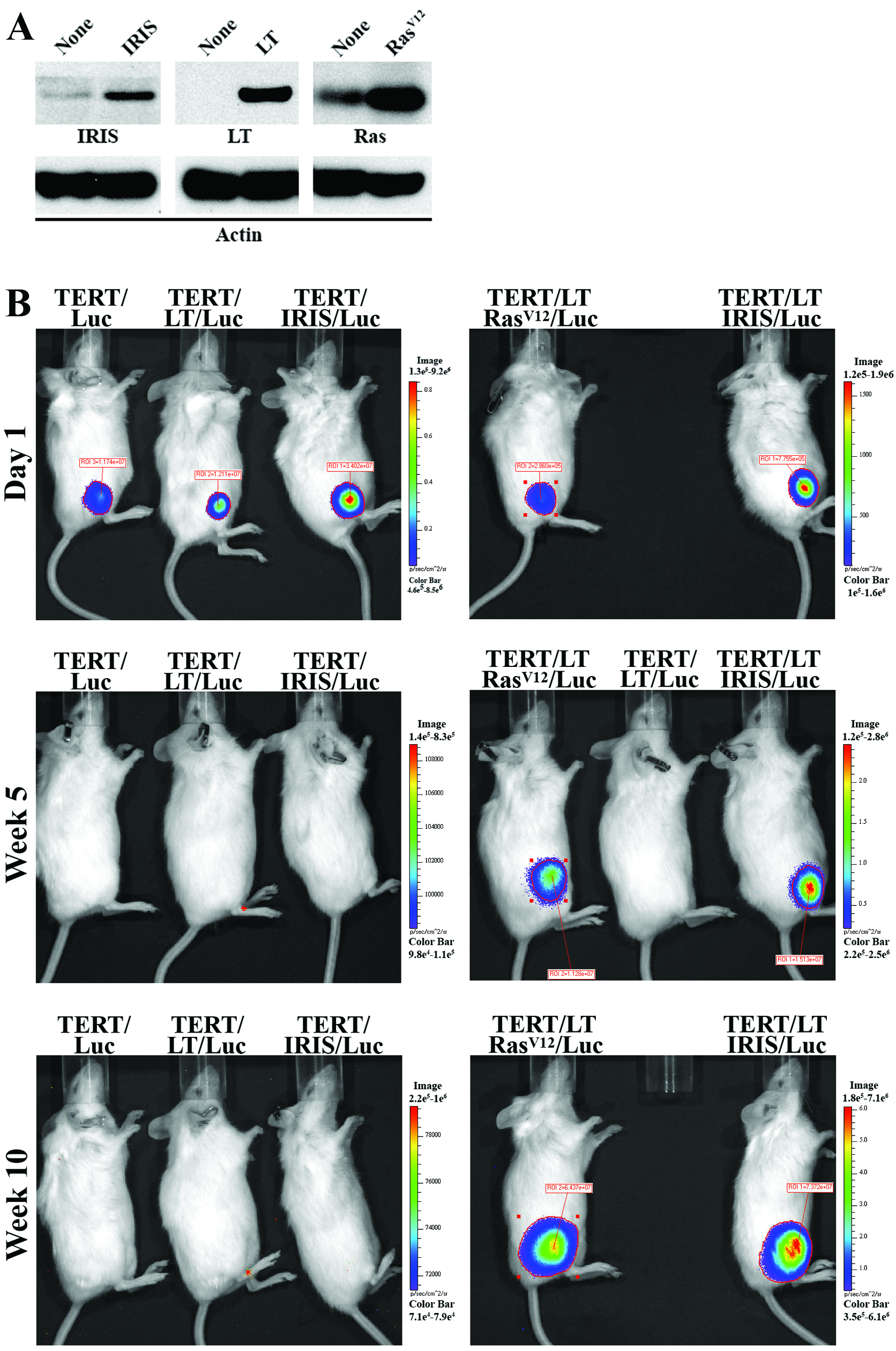

Supplement: Figure S2 — Subcutaneous tumor formation using HME cells expressing TERT/LT/RasV12 or /BRCA1-IRIS. (A) Representative images of luciferase signals in mice injected with HME/Luc cells expressing TERT, TERT/LT, TERT/IRIS, TERT/LT/RasV12, or TERT/LT/IRIS mixed with matrigel at day 1, week 5 or week 10 following cell injection. (TIF) [file pone.0034102.s002.tif]

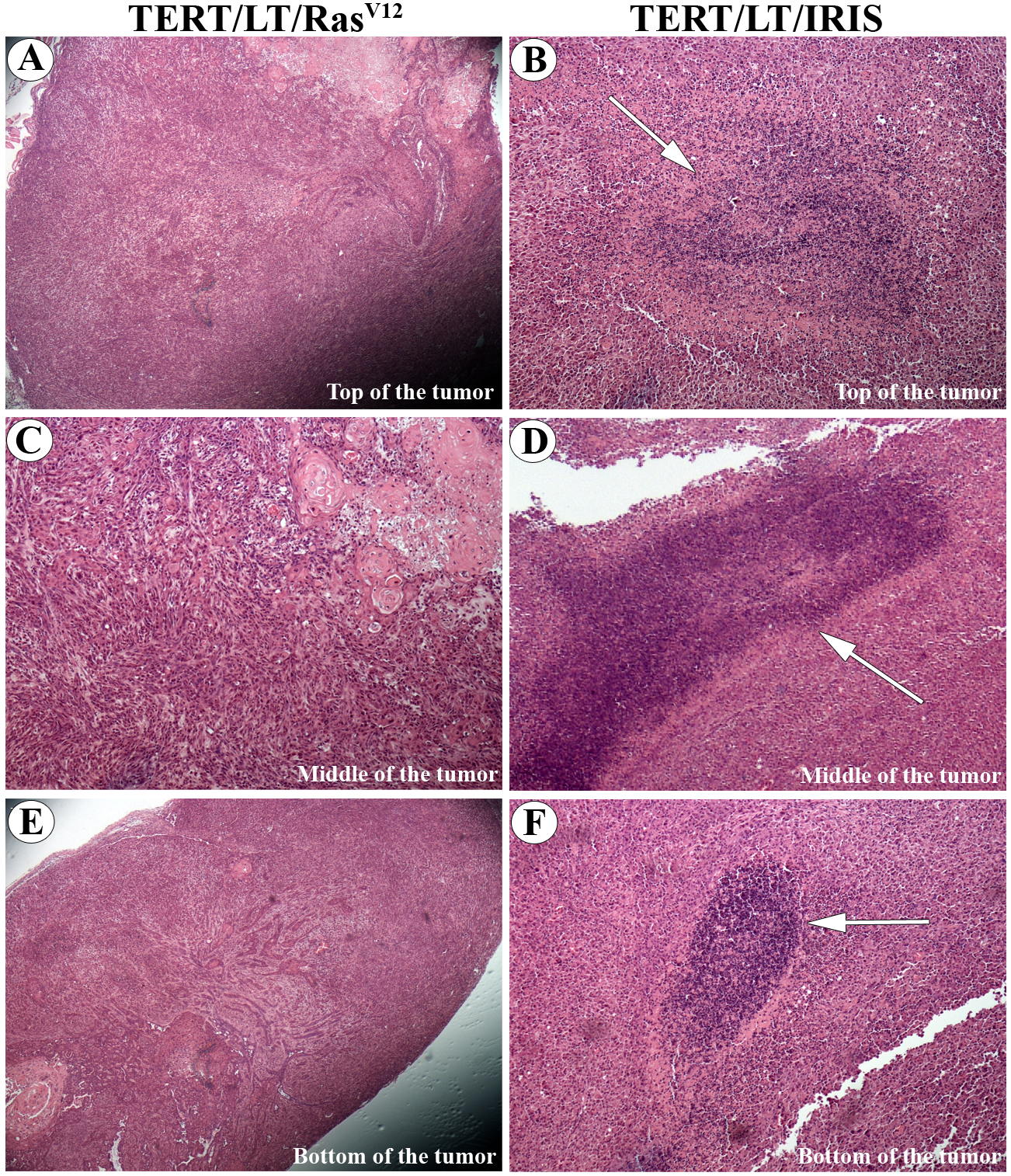

Supplement: Figure S3 — Pronounced necrosis in BRCA1-IRIS-induced and not RasV12-induced tumors. (A, C and E) are sections at different levels; top (A), middle (C) and bottom (E) of a RasV12-induced tumor. (B, D and F) are sections at different levels; top (B), middle (D) and bottom (E) of BRCA1-IRIS induced tumor. Note the pronounced necrosis at all levels in BRCA1-IRIS- (arrows in B, D and F) and not RasV12-induced tumors. (TIF) [file pone.0034102.s003.tif]

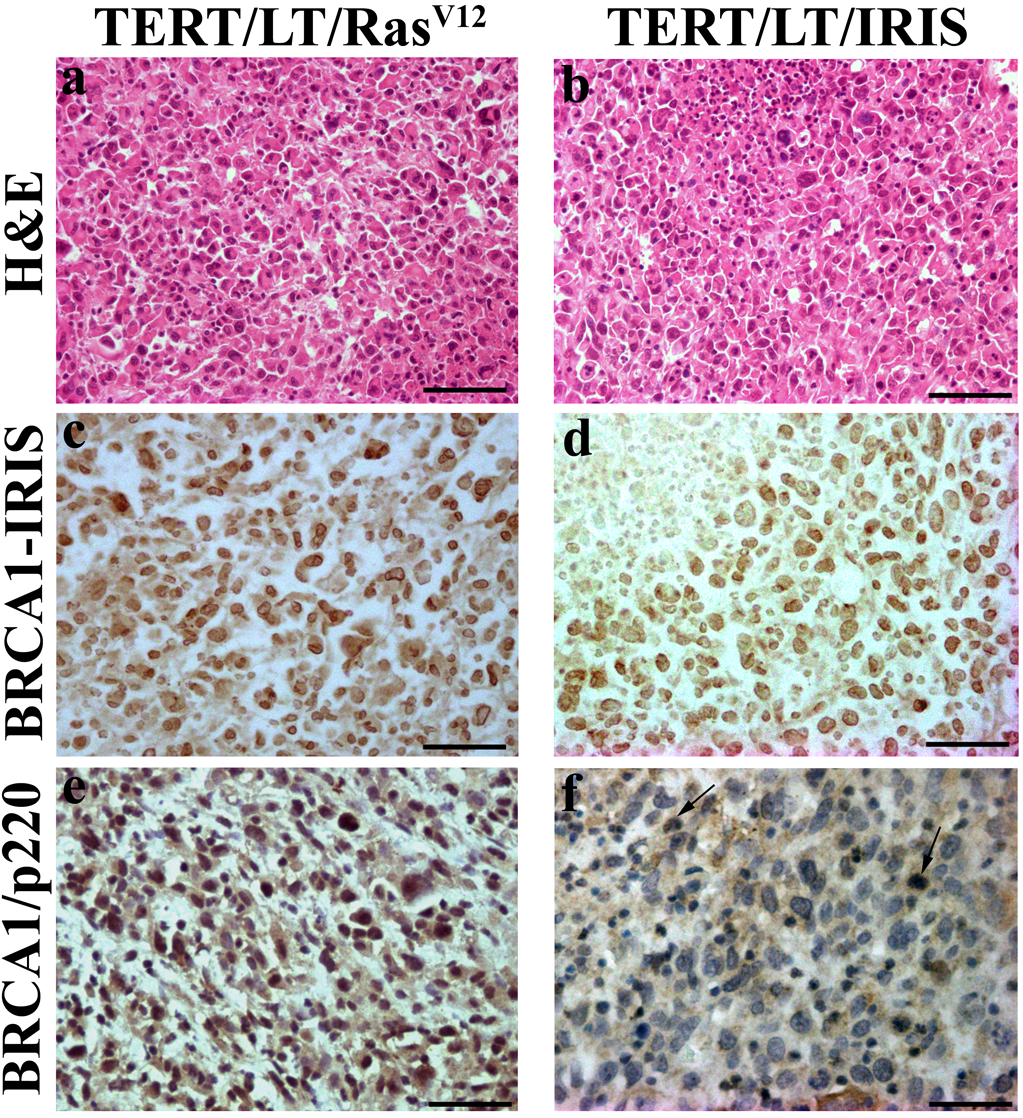

Supplement: Figure S4 — Loss of BRCA1/p220 expression in BRCA1-IRIS- and not RasV12-induced tumors. Representative sections from RasV12- (a, c, and e) or BRCA1-IRIS- (b, d, and f) induced tumors stained with H&E (a and b), for BRCA1-IRIS (c and d) or BRCA1/p220 (e and f). (TIF) [file pone.0034102.s004.tif]

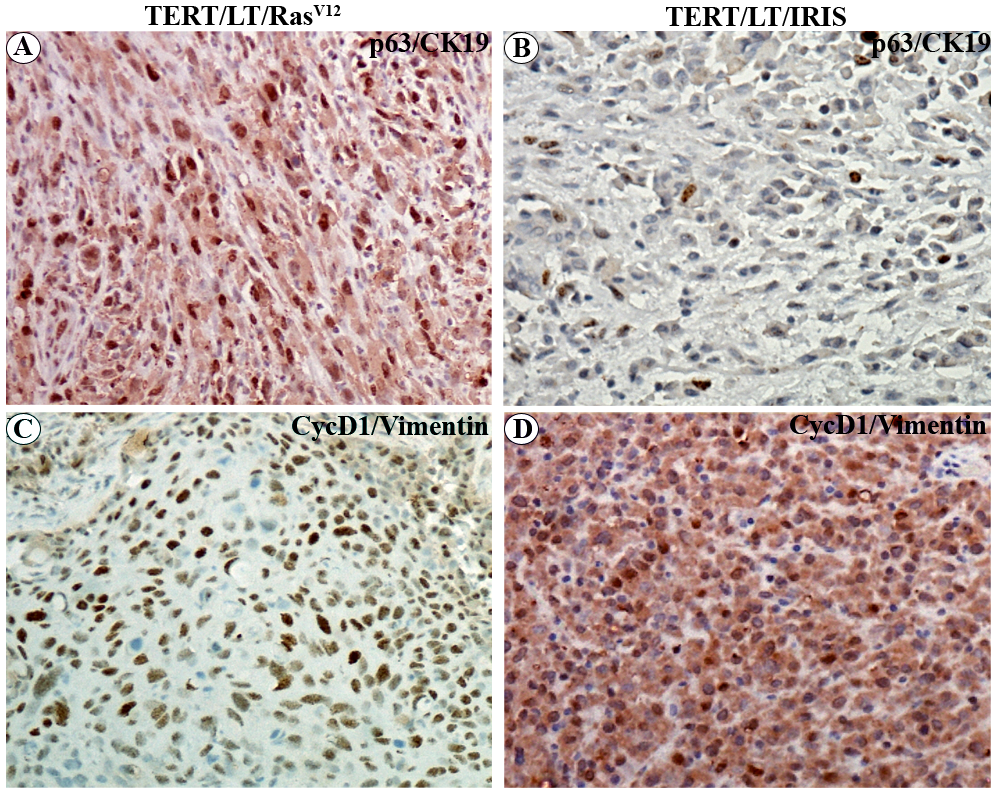

Supplement: Figure S5 — Loss of epithelial marker and gain of mesenchymal marker expression only in BRCA1-IRIS-induced tumors. Representative sections from RasV12- (A and C) or BRCA1-IRIS- (B and D) induced tumors double stained for p63 and cytokeratin (CK) 19 (A and B) or cyclin (Cyc) D1, and vimentin (C and D). (TIF) [file pone.0034102.s005.tif]
